# *Syzygium jambos* Displayed Antibacterial and Antibiotic-Modulating Activities against Resistant Phenotypes

**DOI:** 10.1155/2018/5124735

**Published:** 2018-03-07

**Authors:** Brice E. N. Wamba, Paul Nayim, Armelle T. Mbaveng, Igor K. Voukeng, Joachim K. Dzotam, Ornella J. T. Ngalani, Victor Kuete

**Affiliations:** Department of Biochemistry, Faculty of Science, University of Dschang, Dschang, Cameroon

## Abstract

The present study was designed to evaluate the antibacterial activities of methanol extracts of bark and leaves of* Syzygium jambos*, as well as their synergistic effects with selected antibiotics against drug-resistant Gram-positive and Gram-negative bacteria. The crude extracts were subjected to qualitative phytochemical screening; broth microdilution method was used for antibacterial assays. Phytochemical studies indicate that leaves and bark extracts contained polyphenols, anthraquinones, tannins, and steroids. Extract of the leaves was active against all the 26 strains of* Staphylococcus aureus *and all the 21 strains of Gram-negative bacteria tested, within the minimum inhibitory concentration (MIC) range of 32–512 *μ*g/mL. The lowest MIC value of 32 *μ*g/mL was obtained with extract of the leaves against* Staphylococcus aureus *MRSA9 strain. In Gram-negative bacteria, the lowest MIC value of 64 *μ*g/mL was also obtained against* Enterobacter aerogenes* EA294 and* Klebsiella pneumoniae* K24 strains. Against* S. aureus *strains, antibiotic-modulating activity of extracts at MIC/2 towards more than 70% of the tested strains was obtained when leaves and bark extracts were tested in association with chloramphenicol (CHL). This was also the case when leaves extract was combined with CHL, kanamycin (KAN), tetracycline (TET), and erythromycin (ERY) and when bark extract was combined with ciprofloxacin (CIP), TET, and ERY against Gram-negative bacteria. In conclusion, this study demonstrated that* Syzygium jambos* has antibacterial and antibiotic-modulating activities.

## 1. Introduction

Infectious diseases cause 15 million deaths every year, accounting for about 27.12% of deaths worldwide [1]. Multidrug-resistant (MDR) bacteria are responsible for therapeutic failures, leading to an increase disease burden [2]. Despite the various technological and medical-pharmaceutical advances, MDR bacteria still remain a major cause of morbidity and mortality globally. The search for new antibacterial substances should therefore take into account the development of resistance by pathogenic bacteria. With regard to the high diversity of secondary metabolites in plant kingdom, botanicals constitute a good reservoir for drug discovery to combat MDR bacteria [3, 4]. Also, the loss of efficacy of several antibiotics and the scarcity of new antibacterial agents propel the search for substances capable of restoring the activity of antibiotics. African medicinal plants have previously shown efficiency against MDR bacteria with some of them being able to modulate the activity of antibiotics. Some of these plants include* Xanthosoma mafaffa, Moringa oleifera, Passiflora edulis* [5],* Anthocleista schweinfurthii, Nauclea latifolia, Zehneria scabra *[6],* Nauclea pobeguinii* [7],* Catharanthus roseus, Croton macrostachys, Paullinia pinnata *[8],* Albizia adianthifolia, Alchornea laxiflora, Laportea ovalifolia* [9],* Mangifera indica* [10],* Ricinodendron heudelotii* [11], and* Euphorbia prostrata* [12]. In our continuous search for new botanicals to combat MDR bacteria as well as potentiate the activity of antibiotics, we targeted another African plant,* Syzygium jambos* (L.) Alston. (Myrtaceae). This plant is used traditionally to treat abdominal pain, diarrhea, amenorrhea, pernicious attacks [13], epilepsy, asthma, bronchitis, diuretics, rheumatism, smallpox and eye irritation [14], respiratory disorders, eczema, malaria, and infectious diseases [15]. Previous studies have reported the antibacterial effects of extracts of bark, leaves, and seeds of* Syzygium jambos *against sensitive phenotypes [14]. The present study was aimed at evaluating the antibacterial effects of this plant against resistant phenotypes as well as its ability to reverse the antibiotic resistance. The antibiotic-modulating effect of this plant is being reported for the first time.

## 2. Material and Methods

### 2.1. Plant Material and Extraction

The leaves and bark of* Syzygium jambos* (L.) Alston. (Myrtaceae) were collected in Dschang, Western Region of Cameroon, in April 2016. The plant was identified at the National Herbarium in Yaoundé (Cameroon) where the voucher specimen was conserved under the registration number 30458/HNC. The dried and powdered material (100 g) was macerated in 300 mL of methanol at room temperature for 48 h and then filtered using Whatman filter paper number 1. The filtrate obtained was concentrated using a rotary evaporator under reduced pressure to obtain the crude methanol extract, which was kept at 4°C until further use.

### 2.2. Chemicals

Eight reference antibiotics (RA) purchased from Sigma-Aldrich (St Quentin Fallavier, France) were tested: ampicillin (AMP), cefepime (CEF), chloramphenicol (CHL), ciprofloxacin (CIP), erythromycin (ERY), kanamycin (KAN), streptomycin (STR), and tetracycline (TET). *p*-Iodonitrotetrazolium chloride (INT) (Sigma-Aldrich) was used as bacterial growth revelator; dimethylsulfoxide (DMSO) was used to dissolve the plant extracts.

### 2.3. Bacteria, Culture Media, and Growth Conditions

The tested bacteria included various strains of a Gram-positive bacterium,* Staphylococcus aureus*, and a panel of Gram-negative bacteria. The strains of* Staphylococcus aureus *used were as follows: a reference strain obtained from American Type Culture Collection (ATCC) (ATCC 25923), 1 methicillin-sensitive* S. aureus (*MSSA1), 7 methicillin-resistant* S. aureus *(MRSA) strains (MRSA3, MRSA4, MRSA6, MRSA8, MRSA9, MRSA11, MRSA12) (obtained from the culture collection of the Laboratory of Microbiology, Graduate School of Pharmaceutical Sciences, The University of Tokyo, Japan, and provided by Dr. Dzoyem of the University of Dschang) [16, 17], and 17 resistant clinical laboratory strains of* S. aureus *(SA01, SA07, SA18, SA23, SA36, SA39, SA56, SA64, SA68, SA88, SA114, SA116, SA124, SA126, SA127, SA135, SA139) available in our Laboratory collection and previously isolated from patients in* Ad-Lucem* Hospital in Banka-Bafang (West Region of Cameroon) [18]. Gram-negative bacteria included MDR isolates (laboratory collection) and reference strains of* Escherichia coli* (ATCC8739, AG100, AG100ATet, AG102, MC4100),* Enterobacter aerogenes* (ATCC13048, CM64, EA3, EA27, EA289, EA298, EA294),* Klebsiella pneumoniae* (ATCC11296, KP55, KP63, K24),* Enterobacter cloacae* (ECCI69),* Pseudomonas aeruginosa* (PA01, PA124), and* Providencia stuartii* (NEA16, PS299645). The clinical strains were the laboratory collection from UMR-MD1, University of Marseille, France. Their bacterial features are reported in Tables S1 and S2 (Supplementary Materials). The microorganisms were cultured overnight on Mueller Hinton Agar 24 h prior to any assay. The Mueller Hinton Broth (MHB) was used as liquid culture medium for susceptibility assays.

### 2.4. Preliminary Phytochemical Screenings

Potential classes of antibacterial secondary metabolites such as alkaloids (Dragendorff's and Mayer's tests); terpenoids: sterols (Salkowski's test), saponins (Foam test), and triterpenes (Liebermann–Burchard test); and phenolics: anthraquinones (Borntrager's test), flavonoids (Aluminum chloride test), polyphenols (Ferric chloride test), and tannins (Gelatin test) (Table 3) were investigated according to described phytochemical methods [4, 19].

### 2.5. INT Colorimetric Assay for MIC and Minimum Bactericidal Concentration (MBC) Determinations

The MIC and minimum bactericidal concentration (MBC) determinations on bacteria were performed using the rapid INT colorimetric assay [20] with some modifications as previously described [21]. Samples were dissolved in DMSO/MHB. The final concentration of DMSO was lower than 2.5%. The twofold dilutions of samples were made in 96-well microplates and the tested bacterial concentration was 1.5 × 10^6^ colony forming unit (CFU)/mL. The microplates were incubated at 37°C for 18 h. All assays were in triplicate and repeated thrice. Wells containing MHB, 100 *μ*L of inoculum, and DMSO to a final concentration of 2.5% served as negative control. The MIC of each sample was detected after 18 h incubation at 37°C, following addition (40 *μ*L) of 0.2 mg/mL of INT and incubation at 37°C for 30 minutes as the lowest sample concentration that prevented the color change of the medium and exhibited complete inhibition of microbial growth [20]. The MBC was determined by adding 50 *μ*L aliquots of the preparations, which did not show any growth after incubation during MIC assays, to 150 *μ*L of MHB. These preparations were further incubated at 37°C for 48 h. The MBC was regarded as the lowest concentration of a sample, which did not induce a color change after addition of INT as mentioned above [21].

### 2.6. Antibiotic-Activity Modulation Assays

To evaluate the antibiotic-resistance modulating activity of extracts, a preliminary assay was performed to determine the MICs of antibiotics in the absence and presence of these extracts using broth microdilution method as previously described [20–22].* S. aureus *SA88 and* P. aeruginosa *PA124 were used for preliminary assays and samples were tested at various subinhibitory concentrations (MIC/2, MIC/4, MIC/8, and MIC/16). Results allowed selecting MIC/2 and MIC/4 as subinhibitory concentrations for further experiments on selected* S. aureus *strains as well as Gram-negative bacteria. Briefly, after serial dilution of antibiotic, extract was added to each well at its subinhibitory concentration and the bacterial inoculation was done; the MIC was further determined. Rows receiving antibiotic dilutions without extracts were used for the determination of the MICs of the antibiotics. The modulation factor was defined as the ratio of the MIC of antibiotic alone versus that of antibiotic in the presence of extract. Modulation factor ≥ 2 was set as the cut-off for biologically significance of antibiotic-resistance modulating effects [23].

## 3. Results

### 3.1. Phytochemical Composition of Plant Extracts

The major classes of phytochemicals in the leaves and bark extracts from* Syzygium jambos *were assessed and the results are summarized in Table 1. Both leaves and bark extracts contained polyphenols, anthraquinones, tannins, and steroids whilst alkaloids and flavonoids were absent. Triterpenes and saponins were found only in the bark extract.

### 3.2. Antibacterial Activity

The antibacterial activity of leaves, bark extracts, and CIP against 26 strains of* S. aureus* (Table 2) or CHL against 21 Gram-negative bacteria (Table 3) was determined. Results showed that leaves extract was active against all the strains of* S. aureus *and Gram-negative bacteria within the MIC range of 32–512 *μ*g/mL. The bark extract had selective activity, with MIC values below or equal to 1024 *μ*g/mL being obtained on 22/26 (84.6%) strains of* S. aureus *(Table 2) and 10/21 (47.6%) strains of Gram-negative bacteria (Table 3). The lowest MIC value of 32 *μ*g/mL was noted with the leaves extract against* S. aureus *MRSA9 strain. In Gram-negative bacteria, the lowest MIC value of 64 *μ*g/mL was obtained against* Enterobacter aerogenes* EA294 and* Klebsiella pneumoniae* K24 strains. The MICs of RA were below 4 *μ*g/mL for CIP against* S. aureus *strains and between 4 and 128 *μ*g/mL for CHL against Gram-negative bacteria. MBC values in the range of 128–1024 *μ*g/mL were recorded with leaves extract against all 26 tested* S. aureus *strains and against 17/21 (33.3%) strains of Gram-negative bacteria. The MBC/MIC ratios generally ranged from 2 to 8 for the leaves extract on tested bacteria. However, with bark extract, no recordable MBC value was noted against* S. aureus* strains whilst it was detected against two Gram-negative bacteria (Table 3).

### 3.3. Antibiotic-Resistance Modulation Activity of Extracts

Leaves and bark extracts at MIC/2, MIC/4, MIC/8, and MIC/16 were first tested in combination with 8 antibiotics: CHL, TET, CIP, AMP, CEF, ERY, STR, and KAN against* S. aureus *SA88 and* P. aeruginosa *PA124 strains (Table 4). It appears that the best antibiotic-modulation effects were obtained with the two extracts at MIC/2 and MIC/4. In effect, at MIC/2 and MIC/4 of leaves extract, 2-fold or more increases in antibiotic activities were obtained with 5/8 and 6/8 tested antibiotics, respectively, against* S. aureus *SA88 and with 6/8 and 7/8 tested antibiotics, respectively, against* P. aeruginosa *PA124. Better increases in antibiotic activities were also obtained with bark extract at MIC/2 and MIC/4. Consequently, the two extracts were further tested in combination with the above antibiotics against the reference strains (ATCC 25923), 8 resistant strains of* S. aureus,* and 10 Gram-negative bacteria, at MIC/2 and MIC/4 (Tables 5–8). Results showed that 2-fold or more increases of the activity of antibiotics against more than 70% tested strains of* S. aureus *were obtained when leaves and bark extracts were combined with CHL at MIC/2 (77.78% and 88.89%, resp.) (Tables 5 and 6). Corresponding results against Gram-negative bacteria were also obtained when leaves extract was combined with CHL (90% and 80% at MIC/2 and MIC/4, resp.), KAN at MIC/2 (80%), TET (80% at MIC/2 and MIC/4), and ERY at MIC/2 (80%) (Table 7). This was also the case when bark extract was combined with CIP and TET at MIC/2 (70%) and with ERY (80% and 70 at MIC/2 and MIC/4, resp.) (Table 8).

## 4. Discussion

### 4.1. Phytochemical Composition of Extracts

Polyphenols, anthraquinones, tannins, and steroids were detected leaves and bark extracts (Table 1). The role of several molecules belonging to polyphenols as antibacterials has been demonstrated [3, 4, 24]. Tannins and anthraquinones also belong to a class of polyphenols and their presence in the two extracts could in part explain their antibacterial effects [3]. Previous phytochemical studies of the bark of this plant led to the isolation of triterpenoids such friedelin, *β*-amyrin acetate, betulinic acid, and lupeol from the bark of the plant [25]. This consolidates the presence of triterpenoids in the investigated bark extract.

### 4.2. Antibacterial Potential of Extracts

Overcoming the bacterial resistance to antibiotics is a major challenge in the treatment of infectious diseases. The scarcity of new antibacterials to fight resistant pathogens propels the search for new agents from natural sources. The activity of newly discovered chemotherapeutic agents should take into account the ability of bacteria to rapidly develop resistant phenotypes. In this study, clinical strains of* S. aureus *as well as several Gram-negative bacteria tested were previously reported as resistant to at least one commonly used antibiotic [16–18, 26–30] (Tables S1 and S2). With regard to the diversity of plant secondary metabolites, their use as tools for antibacterial drug discovery is an attractive strategy. According to established criteria, MIC values in the range of 100–1000 *μ*g/mL are indication that botanicals have antimicrobial activities [31]. Also, the antibacterial activity of plant extracts is considered significant if MIC values are below 100 *μ*g/mL, moderate if 100 ≤ MICs ≤ 625 *μ*g/mL, and weak if MICs > 625 *μ*g/mL [32]. Leaves extracts had MIC values below 100 *μ*g/mL against 8/26 tested* S. aureus *strains (Table 2). This clearly indicates that leaves extract of* Syzygium jambos* has good antistaphylococcal potential. Besides, MICs below 100 *μ*g/mL were also obtained with this extract against two tested Gram-negative bacteria (Table 3), confirming the interesting antibacterial potential of the leaves extract contrary to the bark extract. These data are in accordance with previous antibacterial investigations of this plant. In effect, aqueous and acetone extract of the bark, leaves, and seeds of* Syzygium jambos *previously displayed antibacterial effects against sensitive strains of* Staphylococcus aureus, Bacillus subtilis, Enterococcus gallinarum, Escherichia coli, Pseudomonas aeruginosa, Klebsiella pneumoniae, Proteus vulgaris, Enterococcus faecium, Salmonella typhi, *and* Vibrio cholera *[14, 33]. Also, the methanol extract of leaves had antimicrobial activity against* Alcaligenes faecalis, Bacillus subtilis, Staphylococcus aureus, *and* Aeromonas hydrophilia* [34]. The present study focused on resistant phenotypes and therefore provides additional information on the good antibacterial activity of this plant and the ability of the leaves methanol extract to combat resistant phenotypes.

### 4.3. Antibiotic-Modulation Effects of Extracts

The ability of several botanicals and phytochemicals to modulate the antibiotic resistance has been reported [21, 23, 35]. Products able to potentiate the activity of antibiotics on more than 70% of bacteria have been suggested as potential efflux pumps inhibitors [36]. In this study, antibiotic-modulating activity of extracts at MIC/2 on more than 70% tested strains of* S. aureus *was obtained with the association leaves and bark extracts and CHL (Tables 5 and 6). This was also the case with the combination of leaves extract with CHL, KAN, TET, and ERY (Table 7) as well as that of bark extract and CIP, TET, and ERY (Table 8) against Gram-negative bacteria. Consequently, the tested extracts and mostly the leaves extract can be explored more as potential efflux pump inhibitors [36]. To the best of our knowledge, the present study reports for the first time the ability of extracts from* Syzygium jambos *to modulate the activity of antibiotics towards resistant bacteria. It shows that this plant could be used in combination with some antibiotics to combat bacterial resistance to antibiotics. This is in accordance with previous studies on Cameroonian plants such as* Brassica oleracea* var.* botrytis, Brassica oleracea *var.* italica, Capsicum frutescens* var.* fasciculatum, *and* Basilicum polystachyon* which showed synergistic effects with a panel of antibiotics and MDR Gram-negative bacteria tested herein [23].

## 5. Limitations

Our study has limitations. It mainly reports the activity of crude plant extracts, and the identification of the active constituents of the plant would be necessary for better understanding of the reported effects. The toxicity of this plant also needs to be performed to evaluate its safety.

## 6. Conclusion

In the present study, the ability of* Syzygium jambos *and mostly the leaves methanol extract to fight resistant strains of* Staphylococcus aureus *as well as Gram-negative bacteria was demonstrated. It was also found that both leaves and bark extracts could be used as antibiotics resistance modulators, providing a new alternative in the fight against bacterial infections involving resistant phenotypes.

## Figures and Tables

**Table 1:** Extraction yields and phytochemical composition of the plant extracts of *Syzygium jambos*.

Phytochemical classes	*Plant parts, yield (%), and composition*	
	Bark	Leaves
*Yields (%)*	8.2	21.2
Alkaloids	−	−
Polyphenols	+	+
Flavonoids	−	−
Anthraquinones	+	+
Tannins	+	+
Triterpenes	+	−
Steroids	+	+
Saponins	+	−

(−): absent; (+): present; yield calculated as the ratio of the mass of the obtained methanol extract/mass of the plant powder.

**Table 2:** MIC and MBC (in *μ*g/mL) of extracts from *Syzygium jambos* and ciprofloxacin against *Staphylococcus aureus* strains.

Staphylococcus aureus strains	Tested sample, MIC, and MBC in *μ*g/mL and ratio MBC/MIC								
	Leaves extract			Bark extract			Ciprofloxacin		
	MIC	MBC	MBC/MIC	MIC	MBC	MBC/MIC	MIC	MBC	MBC/MIC
*ATCC25923*	**64**	512	8	1024	-	-	**<0.5**	16	<32
*SA01*	256	512	2	-	-	-	**<0.5**	4	<8
*SA07*	128	1024	8	-	-	-	**<0.5**	1	<2
*SA18*	**64**	512	8	1024	-	-	**<0.5**	8	<16
*SA23*	256	1024	4	512	-	-	**<0.5**	<0.5	1
*SA36*	256	256	1	512	-	-	**1**	8	8
*SA39*	128	1024	8	1024	-	-	**<0.5**	16	<32
*SA56*	256	512	2	1024	-	-	**<0.5**	4	<8
*SA64*	128	512	4	1024	-	-	**4**	8	2
*SA68*	128	1024	8	1024	-	-	**<0.5**	<0.5	1
*SA88*	512	1024	2	1024	-	-	**<0.5**	2	<4
*SA114*	128	1024	8	1024	-	-	**<0.5**	<0.5	1
*SA116*	128	1024	8	1024	-	-	**<0.5**	<0.5	1
*SA124*	512	512	1	1024	-	-	**<0.5**	<0.5	1
*SA126*	128	512	4	-	-	-	**<0.5**	<0.5	1
*SA127*	256	512	2	1024	-	-	**<0.5**	<0.5	1
*SA135*	128	512	4	1024	-	-	**<0.5**	1	<2
*SA139*	256	1024	4	-	-	-	**<0.5**	<0.5	1
*MSSA1*	128	512	4	1024	-	-	**2**	16	8
*MRSA3*	**64**	512	8	1024	-	-	**2**	16	8
*MRSA4*	**64**	128	2	256	-	-	**1**	16	16
*MRSA6*	**64**	128	2	1024	-	-	**2**	8	4
*MRSA8*	128	512	4	1024	-	-	**2**	8	4
*MRSA9*	**32**	128	4	512	-	-	**2**	16	8
*MRSA11*	**64**	256	4	512	-	-	**2**	16	8
*MRSA12*	**64**	512	8	1024	-	-	**2**	4	2

MBC/MIC; (-): >1024; MIC value in bold: significant activity.

**Table 3:** MIC and MBC (in *μ*g/mL) of extracts from *Syzygium jambos* and chloramphenicol against Gram-negative bacterial strains.

Bacterial strains	Tested sample, MIC, and MBC in *μ*g/mL and ratio MBC/MIC								
	Leaves extract			Bark extract			Chloramphenicol		
	MIC	MBC	MBC/MIC	MIC	MBC	MBC/MIC	MIC	MBC	MBC/MIC
*Escherichia coli*									
ATTC8739	512	1024	2	-	-	-	**8**	64	8
ATCC10536	128	1024	8	-	-	-	**4**	16	4
AG100	512	1024	2	512	512	1	32	64	2
AG102	512	1024	2	-	-	-	32	256	8
AG100ATet	256	512	2	-	-	-	**4**	32	8
MC4100	512	1024	2	-	-	-	128	-	-
W3110	512	1024	2	-	-	-	**8**	32	4
*Enterobacter aerogenes*									
ATCC13048	256	1024	4	512	-	-	**8**	128	16
EA27	256	512	2	1024	-	-	128	256	2
EA289	512	1024	2	512	1024	2	**4**	64	16
EA294	**64**	512	8	512	-	-	**2**	256	128
EA 298	256	1024	4	-	-	-	**8**	128	16
*Klebsiella pneumoniae*									
ATCC11296	256	512	2	-	-	-	**8**	256	32
K24	**64**	-	-	-	-	-	16	128	8
KP55	256	1024	4	512	-	-	64	128	2
KP63	128	1024	8	-	-	-	16	128	8
*Providencia stuartii*									
PS2636	256	1024	4	1024	-	-	64	256	4
NEA16	128	1024	8	512	-	-	64	128	2
*Enterobacter cloacae*									
ECCI69	512	-	-	512	-	-	128	-	-
*Pseudomonas aeruginosa*									
PA01	512	-	-	512	-	-	128	-	-
PA124	512	-	-	-	-	-	32	-	-

MBC/MIC; (-): >1024 *μ*g/mL; MIC value in bold: significant activity.

**Table 4:** Preliminary antibiotic resistance modulatory activity of extracts at subinhibitory concentrations against *S. aureus ST88 *and *P. aeruginosa *PA124 strains.

Plant extracts and bacterial strains	Extract concentrations	Antibiotics, minimum inhibitory concentration (*μ*g/mL), and fold increase (in brackets)							
		CHL	TET	CIP	AMP	CEF	ERY	STR	KAN
*S. aureus SA88*	0	256	-	2	-	-	32	8	4
Leaves	CMI/2	64 **(4)**	32** (>4)**	1** (2)**	16 **(>16)**	8** (>32)**	16 **(2)**	4 **(2)**	4 (1)
	CMI/4	128 **(2)**	32** (>4)**	1** (2)**	256** (>2)**	32** (>8)**	64 (0.5)	4 **(2)**	4 (1)
	CMI/8	128 **(2)**	-	1** (2)**	256 **(>2)**	-	64 (0.5)	4 **(2)**	4 (1)
	CMI/16	128 **(2)**	-	1** (2)**	-	-	64 (0.5)	4 **(2)**	4 (1)
Bark	CMI/2	32** (8)**	64** (>2)**	≤0.5 **(≥4)**	-	-	4** (8)**	≤2 **(≥4)**	4 (1)
	CMI/4	32** (8)**	64** (>2)**	≤0.5 **(≥4)**	-	-	4** (8)**	≤2 **(≥4)**	4 (1)
	CMI/8	128 **(2)**	-	1** (2)**	-	-	32 (1)	8 (1)	4 (1)
	CMI/16	128 **(2)**	-	2 (1)	-	-	32 (1)	16 (0.5)	4 (1)
*P. aeruginosa *PA124	0	32	16	16	-	-	32	64	64
Leaves	CMI/2	2 **(16)**	1 **(16)**	4 **(4)**	-	-	8 **(4)**	16** (4)**	32 **(2)**
	CMI/4	4 **(8)**	1 **(16)**	4 **(2)**	-	-	32 (1)	32 (**2**)	32 **(2)**
	CMI/8	4 **(8)**	4 **(4)**	4 **(2)**	-	-	32 (1)	32 **(2)**	32 **(2)**
	CMI/16	4 **(8)**	4 **(4)**	16 (1)	-	-	32 (1)	32 **(2)**	32 **(2)**
Bark	CMI/2	32 (1)	8 **(2)**	4 **(4)**	-	-	16 **(2)**	64 (1)	16 **(4)**
	CMI/4	32 (1)	16 (1)	4 **(4)**	-	-	16 **(2)**	64 (1)	32 **(2)**
	CMI/8	32 (1)	16 (1)	4 **(4)**	-	-	16 (1)	64 (1)	64 (1)
	CMI/16	32 (1)	16 (1)	4 **(4)**	-	-	16 (1)	64 (1)	64 (1)

AMP: ampicillin, CEF: cefepime, CIP: ciprofloxacin, Ery: erythromycin, KAN: kanamycin; STR: streptomycin, TET: tetracycline; (-): >256 *μ*g/mL; fold increase in bold: significant effect.

**Table 5:** Resistance modulating effects of the leaves methanol extract from *Syzygium jambos* at its subinhibitory concentrations on selected *S. aureus *strains.

Antibiotics	Bacterial strains, MIC (*μ*g/mL) of antibiotics in the absence and presence of the extract										
	Extract concentration	ATCC 25923	MRSA3	MRSA4	MRSA9	MRSA11	MRSA12	SA18	SA36	SA64	Antibiotic modulating effect (%)
CIP	0	8	≤0.5	8	≤0.5	1	2	2	4	8	
	MIC/2	≤0.5 **(≥16)**	≤0.5 (na)	4 **(2)**	≤0.5 (na)	≤0.5 **(≥2)**	2 (1)	≤0.5 **(≥4)**	≤0.5 **(≥8)**	8 (1)	55.56
	MIC/4	8 (1)	≤0.5 (na)	16 (0.5)	≤0.5 (na)	≤0.5 **(≥2)**	2 (1)	≤0.5 **(≥4)**	≤0.5 **(≥8)**	8 (1)	33.33
CHL	0	64	64	4	128	128	128	8	128	32	
	MIC/2	≤2 **(≥32)**	32 **(2)**	4 (1)	128 (1)	64 **(2)**	64 **(2)**	≤2 **(≥4)**	≤2 **(≥64)**	16 **(2)**	**77.78**
	MIC/4	64 (1)	64 (1)	4 (1)	128 (1)	64 **(2)**	64 **(2)**	4 **(2)**	≤2 **(≥64)**	32 (1)	44.44
TET	0	≤0.5	32	2	32	64	64	2	1	1	
	MIC/2	≤0.5 (na)	32 (1)	≤0.5 **(≥4)**	32 (1)	32 **(2)**	32 **(2)**	≤0.5 **(≥4)**	≤0.5 **(≥2)**	≤0.5 **(≥2)**	66.67
	MIC/4	≤0.5 (na)	32 (1)	≤0.5 **(≥4)**	32 (1)	32 **(2)**	32 **(2)**	1 **(2)**	≤0.5 **(≥2)**	≤0.5 **(≥2)**	66.67
ERY	0	16	32	16	8	16	16	32	≤2	8	
	MIC/2	≤2 **(≥8)**	8 **(4)**	4 **(4)**	8 (1)	16 (1)	16 (1)	≤2 **(≥16)**	≤2 (na)	≤2 **(≥4)**	55.56
	MIC/4	≤2 **(≥8)**	8 **(4)**	4 **(4)**	8 (1)	16 (1)	32 (0.5)	≤2 **(≥16)**	≤2 (na)	8 (1)	44.44
KAN	0	≤2	8	256	16	16	64	8	≤2	32	
	MIC/2	≤2 (na)	8 (1)	256 (1)	16 (1)	16 (1)	256 (0.25)	≤2 **(≥4)**	≤2 (na)	32 (1)	11.11
	MIC/4	≤2 (na)	8 (1)	256 (1)	16 (1)	16 (1)	256 (0.25)	4 **(2)**	≤2 (na)	32 (1)	11.11
CEF	0	-	-	-	-	-	-	-	32	128	
	MIC/2	-	-	-	-	-	-	-	4 **(8)**	8 **(16)**	22.22
	MIC/4	-	-	-	-	-	-	-	16 **(2)**	64 **(2)**	22.22
STR	0	256	128	64	128	64	64	128	128	16	
	MIC/2	≤2 **(≥128)**	64 **(2)**	64 (1)	64 **(2)**	32 **(2)**	64 (1)	4 **(32)**	≤2 **(≥64)**	16 (1)	66.67
	MIC/4	256 (1)	64 **(2)**	64 (1)	64 **(2)**	64 (1)	64 (1)	4 **(32)**	≤2 **(≥64)**	16 (1)	44.44
AMP	0	-	-	256	-	-	-	128	256	-	
	MIC/2	-	-	256 (1)	-	-	-	128 (1)	≤2 **(≥128)**	-	11.11
	MIC/4	-	-	256 (1)	-	-	-	128 (1)	256 (1)	-	0

TET: tetracycline, KAN: kanamycin, ERY: erythromycin, CHL: chloramphenicol, CIP: ciprofloxacin, AMP: ampicillin, STR: streptomycin, CEF: cefepime; (-): MIC not detected at up to 256 *μ*g/mL; (): modulating factor; na: not applicable; MIC: minimum inhibitory concentration; percentage of antibiotic's modulating effect by the plant extracts; values in bold represent modulating factor ≥ 2 and modulating effect observed on more than 70% of the tested MDR bacteria.

**Table 6:** Resistance modulating effects of the bark methanol extract from *Syzygium jambos* at its subinhibitory concentrations on selected *S. aureus* strains.

Antibiotics	Bacterial strains, MIC (*μ*g/mL) of antibiotics in the absence and presence of the extract										
	Extract concentration	ATCC 25923	MRSA3	MRSA4	MRSA9	MRSA11	MRSA12	SA18	SA36	SA64	Antibiotic modulating effect (%)
CIP	0	8	≤0.5	8	≤0.5	1	2	2	4	8	
	MIC/2	8 (1)	≤0.5 (na)	8 (1)	≤0.5 (na)	1 (1)	2 (1)	2 (1)	2 **(2)**	≤0.5 **(≥16)**	22.22
	MIC/4	8 (1)	≤0.5 (na)	16 (0.5)	≤0.5 (na)	1 (1)	2 (1)	2 (1)	2 **(2)**	≤0.5 **(≥16)**	22.22
CHL	0	64	64	4	128	128	128	8	128	32	
	MIC/2	≤2 **(≥32)**	32 **(2)**	≤2 **(≥2)**	64 **(2)**	128 (1)	64 **(2)**	≤2 **(≥4)**	64 **(2)**	≤2 **(≥16)**	**88.89**
	MIC/4	64 (1)	32 **(2)**	4 (1)	64 **(2)**	128 (1)	64 **(2)**	≤2 **(≥4)**	128 (1)	≤2 **(≥16)**	55.56
TET	0	≤0.5	32	2	32	64	64	2	1	1	
	MIC/2	≤0.5 (na)	32 (1)	≤0.5 **(≥4)**	32 (1)	32 **(2)**	64 (1)	1 **(2)**	1 (1)	≤0.5 **(≥2)**	44.44
	MIC/4	≤0.5 (na)	32 (1)	≤0.5 **(≥4)**	32 (1)	32 **(2)**	64 (1)	1 **(2)**	1 (1)	≤0.5 **(≥2)**	44.44
ERY	0	16	32	16	8	16	16	32	≤2	8	
	MIC/2	≤2 **(≥8)**	16 **(2)**	≤2 **(≥8)**	8 (1)	16 (1)	16 (1)	32 (1)	≤2 (na)	≤2 **(≥4)**	44.44
	MIC/4	≤2 **(≥8)**	16 **(2)**	4 **(4)**	8 (1)	16 (1)	16 (1)	32 (1)	≤2 (na)	≤2 **(≥4)**	44.44
KAN	0	≤2	8	256	16	16	64	8	≤2	32	
	MIC/2	≤2 (na)	4 **(2)**	256 (1)	16 (1)	16 (1)	16 **(4)**	≤2 **(≥4)**	≤2 (na)	≤2 **(≥16)**	44.44
	MIC/4	≤2 (na)	8 (1)	256 (1)	16 (1)	16 (1)	32 **(2)**	≤2 **(≥4)**	≤2 (na)	≤2 **(≥16)**	33.33
CEF	0	-	-	-	-	-	-	-	32	128	
	MIC/2	-	-	-	-	-	-	-	4 **(8)**	8 **(16)**	22.22
	MIC/4	-	-	-	-	-	-	-	16 **(2)**	64 **(2)**	22.22
STR	0	256	128	64	128	64	64	128	128	16	
	MIC/2	64 **(4)**	64 **(2)**	≤2 **(≥32)**	64 **(2)**	64 (1)	64 (1)	64 **(2)**	128 (1)	4 **(4)**	66.67
	MIC/4	256 (1)	64 **(2)**	64 (1)	64 **(2)**	64 (1)	64 (1)	64 **(2)**	128 (1)	4 **(4)**	44.44
AMP	0	-	-	256	-	-	-	128	256	-	
	MIC/2	-	-	256 (1)	-	-	-	128 (1)	≤2 **(≥128)**	-	11.11
	MIC/4	-	-	256 (1)	-	-	-	128 (1)	256 (1)	-	0

TET: tetracycline, KAN: kanamycin, ERY: erythromycin, CHL: chloramphenicol, CIP: ciprofloxacin, AMP: ampicillin, STR: streptomycin, CEF: cefepime; (-): MIC not detected at up to 256 *μ*g/mL; (): modulating factor; na: not applicable; MIC: minimum inhibitory concentration; percentage of antibiotic's modulating effect by the plant extracts; values in bold represent modulating factor ≥ 2 and modulating effect observed on more than 70% of the tested MDR bacteria.

**Table 7:** Resistance modulating effects of the leaves methanol extract from *Syzygium jambos* at its subinhibitory concentrations on selected MDR Gram-negative bacteria.

Antibiotics	Extract concentration	Bacteria, MIC (*μ*g/mL), and modulating factors (in bracket)										Antibiotic modulating effect (%)
		*E. coli*		*E. aerogenes*		*K. pneumoniae*		*P. stuartii*		*P. aeruginosa*		
		AG102	AG100Atet	EA27	EA289	KP55	KP63	PS2636	NEA16	PA01	PA 124	
CHL	0	64	8	64	64	64	64	32	64	64	32	
	MIC/2	<2 **(>32)**	2 **(4)**	32 **(2)**	32 **(2)**	32** (2)**	32** (2)**	32 (1)	16 **(4)**	32 **(2)**	2 **(16)**	**90.00**
	MIC/4	32 **(2)**	4 **(2)**	64 (1)	32 **(2)**	32** (2)**	32** (2)**	32 (1)	32** (2)**	32 **(2)**	4 **(8)**	**80.00**
KAN	0	32	4	16	32	64	64	16	32	16	64	
	MIC/2	8 **(4)**	2 **(2)**	2 **(8)**	16 **(2)**	64 (1)	64 (1)	2 **(8)**	4 **(8)**	8 **(2)**	32 **(2)**	**80.00**
	MIC/4	16** (2)**	4 (1)	2** (8)**	32 (1)	64 (1)	64 (1)	4 **(4)**	16** (2)**	16 (1)	32 **(2)**	50.00
STP	0	128	256	256	64	64	256	256	16	256	64	
	MIC/2	2 **(64)**	2 **(128)**	8** (32)**	64 (1)	32** (2)**	64 **(4)**	256 (1)	16 (1)	256 (1)	16 **(4)**	60.00
	MIC/4	64 **(2)**	128 **(2)**	64** (2)**	64 (1)	32** (2)**	128** (2)**	256 (1)	16 (1)	256 (1)	32 **(2)**	60.00
CIP	0	8	1	1	1	8	4	16	2	2	16	
	MIC/2	8 (1)	<0.5 **(>2)**	1 (1)	1 (1)	8 (1)	2** (2)**	2** (8)**	2 (1)	1 **(2)**	4 **(4)**	50.00
	MIC/4	8 (1)	<0.5 **(>2)**	4 (1)	1 (1)	8 (1)	2** (2)**	8** (2)**	2 (1)	2 (1)	8 **(2)**	40.00
TET	0	8	<0,5	64	32	16	32	4	32	16	16	
	MIC/2	<0.5 **(>16)**	<0,5 (na)	16** (4)**	8 **(4)**	2 **(8)**	16** (2)**	1** (4)**	32 (1)	2 **(8)**	1 **(16)**	**80.00**
	MIC/4	4 **(2)**	<0,5 (na)	16** (4)**	16 **(2)**	2 **(8)**	16** (2)**	1 **(4)**	32 (1)	2 **(8)**	1 **(16)**	**80.00**
ERY	0	64	8	16	64	64	32	16	32	16	32	
	MIC/2	16 **(4)**	2 **(4)**	4** (4)**	32** (2)**	32** (2)**	32 (1)	8** (2)**	16** (2)**	8 **(2)**	8 **(4)**	**90.00**
	MIC/4	16 **(4)**	2 **(4)**	16 (1)	32 (**2)**	64 (1)	32 (1)	8** (2)**	32 (1)	8 **(2)**	32 (1)	50.00

TET: tetracycline, KAN: kanamycin, ERY: erythromycin, CHL: chloramphenicol, CIP: ciprofloxacin, AMP: ampicillin, STR: streptomycin, CEF: cefepime; (-): MIC not detected at up to 256 *μ*g/mL; (): modulating factor; na: not applicable; MIC: minimum inhibitory concentration; percentage of antibiotic's modulating effect by the plant extracts; values in bold represent modulating factor ≥ 2 and modulating effect observed on more than 70% of the tested MDR bacteria.

**Table 8:** Resistance modulating effects of the bark methanol extract from *Syzygium jambos* at its subinhibitory concentrations on selected MDR Gram-negative bacteria.

Antibiotics	Extract concentration	Bacteria, MIC (*μ*g/mL), and modulating factors (in bracket)										Antibiotic modulating effect (%)
		*E. coli*		*E. aerogenes*		*K. pneumoniae*		*P. stuartii*		*P. aeruginosa*		
		AG102	AG100Atet	EA27	EA289	KP55	KP63	PS2636	NEA16	PA01	PA 124	
CHL	0	64	8	64	64	64	64	32	64	64	32	
	MIC/2	16 **(4)**	8 **(4)**	64 (1)	64 (1)	64 (1)	16 **(4)**	8 **(4)**	32** (2)**	64 (1)	32 (1)	50.00
	MIC/4	32 **(2)**	16 **(2)**	64 (1)	64 (1)	64 (1)	32 **(2)**	16 **(2)**	32** (2)**	64 (1)	32 (1)	50.00
KAN	0	32	4	16	32	64	64	16	32	16	64	
	MIC/2	32 (1)	4 (1)	4** (4)**	8 (1)	64 (1)	64 (1)	2 **(4)**	16** (2)**	4 **(4)**	16 **(4)**	50.00
	MIC/4	32 (1)	4 (1)	4** (4)**	8 (1)	64 (1)	64 (1)	4 **(4)**	16 **(2)**	16 (1)	32 **(2)**	40.00
STP	0	128	256	256	64	64	256	256	16	64	64	
	MIC/2	32 **(4)**	64 **(4)**	64 **(4)**	128 (1)	32 **(2)**	256 (1)	256 (1)	8 **(2)**	64 (1)	64 (1)	50.00
	MIC/4	128 (1)	256 (1)	64 **(4)**	128 (1)	32 **(2)**	128 **(2)**	256 (1)	16 (1)	64 (1)	64 (1)	30.00
CIP	0	8	1	1	1	8	4	16	2	2	16	
	MIC/2	8 (1)	<0.5 **(≥2)**	1 (1)	0.5 **(2)**	16 (1)	0,5 **(2)**	<0,5 **(≥32)**	1 **(2)**	0.5 **(4)**	4 **(4)**	**70.00**
	MIC/4	8 (1)	1 (1)	1 (1)	1 (1)	16 (1)	0,5 **(2)**	0,5 **(32)**	1 (1)	1 **(2)**	4 **(4)**	40.00
TET	0	8	<0,5	64	32	16	32	4	32	16	16	
	MIC/2	4 **(2)**	<0,5 (na)	64 (1)	8 **(4)**	16 (1)	4 **(8)**	<0,5 **(≥8)**	2 (**16)**	2 **(8)**	8 **(2)**	**70.00**
	MIC/4	4 **(2)**	<0,5 (na)	64 (1)	8 **(4)**	16 (1)	16 **(2)**	<0,5 **(≥8)**	8 **(4)**	2 (8)	16 (1)	50.00
ERY	0	64	8	16	64	64	32	16	32	16	32	
	MIC/2	4 **(2)**	<0,5 **(≥16)**	64 (1)	8 **(4)**	16 (1)	4 **(8)**	<0,5 **(≥8)**	2 **(16)**	8 **(2)**	16 **(2)**	**80.00**
	MIC/4	4 **(2)**	<0,5 **(≥16)**	64 (1)	8 **(4)**	16 (1)	16 **(2)**	<0,5 **(≥8)**	8 **(4)**	16 (1)	16 **(2)**	**70.00**

TET: tetracycline, KAN: kanamycin, ERY: erythromycin, CHL: chloramphenicol, CIP: ciprofloxacin, AMP: ampicillin STR: streptomycin, CEF: cefepime; (-): MIC not detected at up to 256 *μ*g/mL; (): modulating factor; na: not applicable; MIC: minimum inhibitory concentration; percentage of antibiotic's modulating effect by the plant extracts; values in bold represent modulating factor ≥ 2 and modulating effect observed on more than 70% of the tested MDR bacteria.

## Abbreviations

ATCC: American Type Culture Collection
CEF: Cefepime
CFU: Colony forming unit
CHL: Chloramphenicol
CIP: Ciprofloxacin
DMSO: Dimethylsulfoxide
ERY: Erythromycin
INT: *p*-Iodonitrotetrazolium chloride
KAN: Kanamycin
MBC: Minimum bactericidal concentration
MDR: Multidrug-resistant
MHB: Mueller Hinton Broth
MIC: Minimum inhibitory concentration
MRSA: Methicillin-resistant* Staphylococcus aureus*
RA: Reference antibiotic
SA: * Staphylococcus aureus*
STR: Streptomycin
TET: Tetracycline.

## Supplementary Materials

Supplementary Materials Table S1:* Staphylococcus aureus* strains and features. Table S2: Gram-negative bacteria and features.
