# Supplementary material for: Syzygium jambos Displayed Antibacterial and Antibiotic-Modulating Activities against Resistant Phenotypes
Source: Evid Based Complement Alternat Med. 2018 Mar 7;2018:5124735. doi: 10.1155/2018/5124735 (PMC5863293; doi:10.1155/2018/5124735)
Supplement: Supplementary Materials — Table S1: Staphylococcus aureus strains and features. Table S2: Gram-negative bacteria and features. [file 5124735.f1.pdf]

Sygysium jambos displayed antibacterial and antibiotic-modulating activities against resistant phenotypes

Brice E. N. Wamba[1#], Paul Nayim[1#], Armelle T. Mbaveng[1*], Igor K. Voukeng[1], Joachim K. Dzotam[1], Ornella J.T. Ngalani[1], and Victor Kuete[1**].

[1]*Department of Biochemistry, Faculty of Science, University of Dschang, Cameroon*

#: the two authors contributed equally to the work

Author's addresses

Elvis B. N. Wamba: wambaelvis@yahoo.fr

Paul Nayim: nayimpaul@yahoo.fr
Armelle T. Mbaveng: armkuete@yahoo.fr

Igor Voukeng : Tefogang@yahoo.fr

Joachim K. Dzotam: kamgue_joachim@yahoo.fr

Ornella J.T. Ngalani: ngalaniornella@yahoo.fr

Victor Kuete: kuetevictor@yahoo.fr

Corresponding authors:

⁎ Tel.: +237 675468927; E-mail address: armbatsa@yahoo.fr (Dr A.T. Mbaveng)

*** Tel.: +237 677355927; E-mail address: kuetevictor@yahoo.fr (Prof. Dr Victor Kuete)*

Table S1. ***Staphylococcus aureus*** **strains and features**

**Bacteria**	**Features**	**References**
S. aureus MSSA1	Clinical isolate : Met susceptible ; Nis^r, Chl^r	[1, 2]
S. aureus MRSA3	Clinical isolate : Ofxa^r, Kan^r, Tet^r, Erm^r	[1]
S. aureus MRSA4	Clinical isolate : Ofxa^r, Kan^r, Cyp^r, Chl^r, Gen^r, Nis^r, Amp^r	[1, 2]
S. aureus MRSA6	Clinical isolate : Ofxa^r, Flx^r, Kan^r, Tet^r, Cyp^r, IM/Cs^r, Chl^r, Gen^r, Nis^r, Amp^r	[1, 2]
S. aureus MRSA8	Clinical isolate : Ofxa^r, Flx^r, Kan^r, Erm^r, Cyp^r, Im/Cs^r, Chl^r, Gen^r, Nis^r, Amp^r	[1, 2]
S. aureus MRSA9	Clinical isolate : Ofxa^r, Flx^r, Tet^r, Erm^r, Cyp^r, Im/Cs^r, Chl^r, Gen^r, Nis^r, Amp^r	[1, 2]
S. aureus MRSA11	Clinical isolate : Ofxa^r, Kan^r, Erm^r, Cyp^r, Im/Cs^r, Chl^r, Nis^r, Amp^r	[1, 2]
S. aureus MRSA12	Clinical isolate : Ofxa^r, Flx^r, Kan^r, Erm^r, Im/Cs^r, Chl^r, Gen^r, Nis^r, Amp^r	[1, 2]
ATCC 25923	Reference strain	/
SA01	Clinical isolate : Erm^r, Amp^r	[3]
SA07	Clinical isolate : Erm^r, Dox^r	[3]
SA18	Clinical isolate : Amp^r, Dox^r, Vm^r	[3]
SA23	Clinical isolate : Imi^r, Aug^r	[3]
SA36	Clinical isolate : Dox^r, Vm^r	[3]
SA39	Clinical isolate : Amp^r	[3]
SA56	Clinical isolate : Amp^r, Dox^r	[3]
SA64	Clinical isolate : Amp^r, Dox^r	[3]
SA68	Clinical isolate : Amp^r, Vm^r	[3]
SA88	Clinical isolate : Erm^r, Vm^r	[3]
SA114	Clinical isolate : Amp^r, Dox^r	[3]
SA116	Clinical isolate : Erm^r	[3]
SA124	Clinical isolate : Erm^r	[3]
SA126	Clinical isolate : Amp^r, Dox^r	[3]
SA127	Clinical isolate : Amp^r, Dox^r	[3]
SA135	Clinical isolate : Erm^r	[3]
SA139	Clinical isolate : Erm^r	[3]

Chl^r, Cyp^r, Erm^r, Flx^r, Im/Cs^r, Kan^r, Met^r, Ofxa^r, Tet^r, Vm^r, Amp^r, Dox^r, Aug^r, Gen^r and Nis^r résistance to : chloramphenicol; Cyprofloxacin; Erythromycin; Flomoxef; Imipenem/CilaSAatin sodium; Kanamycin; Méthicillin; Ofloxacin; Tetracycline; Vancomycin ; Ampicillin ; Doxycycline ; Augmentin ; Gentamicin ; Nisin respectively, *SA : SAaphycoccus aureus.*

Table S2. Gram-negative bacteria and features

Strains	Features and References	
Escherichia coli		
ATCC8739	Reference strain	
AG100	Wild-type *E. coli* K-12	[4]
AG100A$_{TET}$	Δ*acrAB* mutant AG100, with over-expressing *acrF* gene ; TET^{R}	[4]
AG102	Δ*acrAB* mutant AG100, owing *acrF* gene markedly over-expressed; TET^{R}	[5, 6]
MC4100	Wild type *E. coli*	[7]
Enterobacter aerogenes		
ATCC13048	Reference strains	
CM64	CHL^{R} resistant variant obtained from ATCC13048 over-expressing the AcrAB pump	[8]
EA3	Clinical MDR isolate; CHL^{R}, NOR^{R}, OFX^{R}, SPX^{R}, MOX^{R}, CFT^{R}, ATM^{R}, FEP^{R}	[9, 10]
EA27	Clinical MDR isolate exhibiting energy-dependent norfloxacin and chloramphenicol efflux with KAN^{R} AMP^{R} NAL^{R} STR^{R} TET^{R}	[9, 10]
EA289	KAN sensitive derivative of EA27	[11]
EA294	EA289 a*crA::*KAN^{R}	[11]
EA298	EA 289 *tolC::*KAN^{R}	[11]
Enterobacter cloacae		
ECCI69	Clinical MDR isolates, CHL^{R}	[12]
Klebsiella pneumoniae		
ATCC12296	Reference strains	
KP55	Clinical MDR isolate, TET^{R} , AMP^{R}, ATM^{R}, CEF^{R}	[13]
KP63	Clinical MDR isolate, TET^{R,} CHL^{R}, AMP^{R,} ATM^{R}	[13]
K24	AcrAB-TolC, Laboratory collection of UNR-MD1, University of Marseille, France	[12]
Providencia stuartii		
NEA16	Clinical MDR isolate, AcrAB-TolC	
PS299645	Clinical MDR isolate, AcrAB-TolC	[14]
Pseudemonas aeruginosa		
PA 01	Reference strains	
PA 124	MDR clinical isolate	[15]

[a]AMP, ATM^{R}, CEF^{R}, CFT^{R}, CHL^{R}, FEP^{R}, KAN^{R}, MOX^{R}, OFX^{R}, STR^{R}, TET^{R}. Resistance to ampicillin, aztreonam, cephalothin, cefadroxil, chloramphenicol, cefepime, kanamycin, moxalactam, ofloxacin, streptomycin, and tetracycline; MDR : Multidrug resistant; AcrAB-TolC efflux pump AcrAB associate to TolC porin,

References

1. A. Paudel, H. Hamamoto, Y. Kobayashi, S. Yokoshima, T. Fukuyama, and K. Sekimizu. "Identification of novel deoxyribofuranosyl indole antimicrobial agents," *J Antibiot (Tokyo), vol.* 65, no. 2, pp. 53-57.
2. J.P. Dzoyem, H. Hamamoto, B. Ngameni, B.T. Ngadjui, and K. Sekimizu. "Antimicrobial action mechanism of flavonoids from *Dorstenia* species," *Drug Discov Ther, vol.* 7, no. 2, pp. 66-72.
3. T.J.O. Ngalani, *[Influence de la multi-résistance des bactéries entériques aux antibiotiques sur l'état immunologique des patients séropositifs au VIH venus en consultation à l'hôpital Adlucem Banka de Bafang],* Department of Biochemistry, University of Dschang, Cameroon, 2015.
4. M. Viveiros, A. Jesus, M. Brito, et al. "Inducement and reversal of tetracycline resistance in Escherichia coli K-12 and expression of proton gradient-dependent multidrug efflux pump genes," *Antimicrob Agents Chemother, vol.* 49, no. 8, pp. 3578-3582.
5. C.A. Elkins and L.B. Mullis. "Substrate competition studies using whole-cell accumulation assays with the major tripartite multidrug efflux pumps of *Escherichia coli*," *Antimicrob Agents Chemother, vol.* 51, no. 3, pp. 923-929.
6. V. Kuete, S. Alibert-Franco, K.O. Eyong, et al. "Antibacterial activity of some natural products against bacteria expressing a multidrug-resistant phenotype," *Int J Antimicrob Agents, vol.* 37, no. 2, pp. 156-161.
7. P. Baglioni, L. Bini, S. Liberatori, V. Pallini, and L. Marri. "Proteome analysis of *Escherichia coli* W3110 expressing an heterologous sigma factor," *Proteomics, vol.* 3, no. 6, pp. 1060-1065.
8. D. Ghisalberti, M. Masi, J.M. Pages, and J. Chevalier. "Chloramphenicol and expression of multidrug efflux pump in *Enterobacter aerogenes*," *Biochem Biophys Res Commun, vol.* 328, no. 4, pp. 1113-1118.
9. M. Mallea, J. Chevalier, C. Bornet, et al. "Porin alteration and active efflux: two in vivo drug resistance strategies used by *Enterobacter aerogenes*," *Microbiology, vol.* 144 (Pt 11), pp. 3003-3009.
10. M. Mallea, A. Mahamoud, J. Chevalier, et al. "Alkylaminoquinolines inhibit the bacterial antibiotic efflux pump in multidrug-resistant clinical isolates," *Biochem J, vol.* 376, no. Pt 3, pp. 801-805.
11. E. Pradel and J.M. Pages. "The AcrAB-TolC efflux pump contributes to multidrug resistance in the nosocomial pathogen *Enterobacter aerogenes,*" *Antimicrob Agents Chemother, vol.* 46, no. 8, pp. 2640-2643.
12. A.G. Fankam, V. Kuete, I.K. Voukeng, J.R. Kuiate, and J.M. Pages. "Antibacterial activities of selected Cameroonian spices and their synergistic effects with antibiotics against multidrug-resistant phenotypes," *BMC Complement Altern Med, vol.* 11, p. 104.
13. J. Chevalier, J.M. Pages, A. Eyraud, and M. Mallea. "Membrane permeability modifications are involved in antibiotic resistance in *Klebsiella pneumoniae*," *Biochem Biophys Res Commun, vol.* 274, no. 2, pp. 496-499.
14. Q.T. Tran, K.R. Mahendran, E. Hajjar, et al. "Implication of porins in beta-lactam resistance of *Providencia stuartii.*," *J Biol Chem, vol.* 285, no. 42, pp. 32273-32281.
15. V. Lorenzi, A. Muselli, A.F. Bernardini, et al. "Geraniol restores antibiotic activities against multidrug-resistant isolates from gram-negative species," *Antimicrob Agents Chemother, vol.* 53, no. 5, pp. 2209-2211.
